# From Menopause to Neurodegeneration—Molecular Basis and Potential Therapy

**DOI:** 10.3390/ijms22168654

**Published:** 2021-08-11

**Authors:** Yu-Jung Cheng, Chieh-Hsin Lin, Hsien-Yuan Lane

**Affiliations:** 1 Department of Physical Therapy and Graduate Institute of Rehabilitation Science, China Medical University, Taichung 40402, Taiwan; chengyu@mail.cmu.edu.tw; 2 Department of Rehabilitation, China Medical University Hospital, Taichung 40402, Taiwan; 3 Institute of Clinical Medical Science, China Medical University, Taichung 40402, Taiwan; 4 Graduate Institute of Biomedical Sciences, China Medical University, Taichung 40402, Taiwan; 5 Kaohsiung Chang Gung Memorial Hospital, Chang Gung University College of Medicine, Kaohsiung 83301, Taiwan; 6 School of Medicine, Chang Gung University, Taoyuan 33302, Taiwan; 7 Department of Psychiatry & Brain Disease Research Center, China Medical University Hospital, Taichung 40402, Taiwan; 8 Department of Psychology, College of Medical and Health Sciences, Asia University, Taichung 41354, Taiwan

**Keywords:** menopause, estrogen, neurodegenerative disease, Alzheimer’s disease, IGF-1, NMDAR

## Abstract

The impacts of menopause on neurodegenerative diseases, especially the changes in steroid hormones, have been well described in cell models, animal models, and humans. However, the therapeutic effects of hormone replacement therapy on postmenopausal women with neurodegenerative diseases remain controversial. The steroid hormones, steroid hormone receptors, and downstream signal pathways in the brain change with aging and contribute to disease progression. Estrogen and progesterone are two steroid hormones which decline in circulation and the brain during menopause. Insulin-like growth factor 1 (IGF-1), which plays an import role in neuroprotection, is rapidly decreased in serum after menopause. Here, we summarize the actions of estrogen, progesterone, and IGF-1 and their signaling pathways in the brain. Since the incidence of Alzheimer’s disease (AD) is higher in women than in men, the associations of steroid hormone changes and AD are emphasized. The signaling pathways and cellular mechanisms for how steroid hormones and IGF-1 provide neuroprotection are also addressed. Finally, the molecular mechanisms of potential estrogen modulation on N-methyl-d-aspartic acid receptors (NMDARs) are also addressed. We provide the viewpoint of why hormone therapy has inconclusive results based on signaling pathways considering their complex response to aging and hormone treatments. Nonetheless, while diagnosable AD may not be treatable by hormone therapy, its preceding stage of mild cognitive impairment may very well be treatable by hormone therapy.

## 1. Introduction

Neurodegenerative diseases include movement, cognitive, and behavioral disorders which are caused along the process of neurodegeneration. The most common neurodegenerative diseases are Alzheimer’s disease (AD), Parkinson’s disease (PD), and dementia with Lewy bodies (DLB) [1,2,3]. Slow progression, lack of definitive diagnosis tools, and complex pathophysiology make these neurodegenerative diseases, especially AD, difficult to treat. In these neurodegenerative diseases, increases in neuronal loss are found with disease progression [4,5,6]. The causes of neuronal loss can be protein misfolding, overloaded proteostasis networks, oxidative stress, programmed cell death, and neuroinflammation [7]. In addition, endocrine dyscrasia by menopause and andropause is also correlated to neuronal dysfunction, cell death, and cognitive decline [8].

Many neurodegenerative diseases display sex-specific features. Pieces of evidence show that aging and female gender are more commonly related with incidence and prevalence of AD but not parkinsonism [9,10]. In addition to incidence and prevalence, the disease progression and treatment responses are also different according to gender [11]. Estrogen and progesterone show protective activity on brain functions, and thus loss of these steroid hormones at menopause is an important risk factor for AD progression [12,13]. In rats, ovariectomy causes a decrease in dendritic spines in hippocampus pyramidal cells, and this reduction can be reversed by 17β-estradiol treatment [14]. The same situation has also been observed in non-human primates. Compared to premenopausal rhesus macaques, rhesus macaques with natural menopause showed worse recognition memory and lower synapse density in the hippocampus [15]. In addition, the frequency of multisynaptic boutons decreased by over 50% after ovariectomy in aged monkeys compared to normal aging monkeys [16].

In this review, we first present evidence showing the sex differences, especially the impacts of menopause, in major neurodegenerative diseases. Then, we review the literature on the possible mechanisms of estrogen, progesterone, and other neurotrophic factors in the progression of neurodegenerative diseases. Finally, we present details on what is known about the molecular pathways through which menopause contributes to neurodegenerative diseases.

## 2. Sex Differences in Major Neurodegenerative Diseases

### 2.1. Alzheimer’s Disease (AD)

Sex-related differences in AD have been well identified in clinical manifestations [17]. Women are more susceptible to AD, and incidence rates are higher in women than in men in Europe and the U.S. [18,19,20,21,22]. Beam and colleagues analyzed 16,926 women and men with any dementia, AD alone, and non-AD dementia alone and found that the incidence rates of all were greater in women [20]. Compared to men, women with AD dementia reach partial loss of autonomy faster, which means they live with more disability longer [23]. Amyloid-beta (Aβ) and hyperphosphorylated tau accumulation are the two major pathological changes in AD progression. Several studies show there is no sex difference in diffuse Aβ plaque deposit in brain or in cerebrospinal fluid (CSF) concentrations [24,25]. Unlike Aβ, higher numbers of neurofibrillary tangles in brains are found in women with AD than in men [24]. PET imaging studies have revealed no gender associated with tau accumulation in aging and early AD [26,27]. Studies which evaluated total tau and phosphorylated tau levels in CSF showed that sex had no impact on patients with AD or mild cognitive impairment (MCI) [28,29].

### 2.2. Parkinson’s Disease (PD)

The prevalence of PD, the second most common neurodegenerative disease, is about 315 per 100,000 persons of all ages [30]. PD is attributed to progressive degeneration of midbrain dopaminergic neurons and motor symptoms. The loss of dopaminergic neurons is sex-related, which has been found in PD animal models and PD patients. Epidemiological studies show that the male sex is one of the risk factors for PD at all ages [22,31,32,33,34,35,36]. Comparing women of similar ages, the incidence and prevalence of PD are higher in postmenopausal than in premenopausal women [37,38,39], which implicates the benefit of estrogen and progesterone on PD onset. However, hormone replacement therapy in women has inconsistent results. A clinical trial on women with Parkinson’s disease with or without dementia showed that estrogen replacement therapy has protective effects for the development of dementia [40]. Another multicenter case–control study showed that oral contraceptives could increase the risk of PD [41]. A meta-analysis showed that estrogen replacement therapy can significantly decrease the risk of onset and/or development of PD [42].

### 2.3. Dementia with Lewy Bodies (DLB)

The gender difference in prevalence of DLB is still inconclusive. A systematic review from 2014 summarized eight studies of DLB and reported that five studies showed disproportionately more females with the disease [43]. Later studies also showed inconsistency in gender distribution. Some studies showed that male sex is more prevalent [44,45,46,47], some favored women [48], and others showed no significance [49]. Mouton et al. investigated gender differences in DLB, AD, PD, and Parkinson’s disease dementia (PDD) from the French national database spanning from 2010 to 2015. The result from a total of 237,695 patients showed that the sex ratio (female percent/male percent) is 1.21 for DLB, 2.34 for AD, 0.76 for PD, and 0.83 for PDD. This large-scale study agrees with the balanced sex distribution in DLB compared with AD and PD–PDD. Regarding biomarkers in the CSF of patients with DLB, women had lower alpha-synuclein (α-syn) CSF Aβ42 levels compared to men, and no sex differences for phospho-tau concentrations were found [50].

## 3. Role of Hormones and Trophic Factors in Neurodegenerative Diseases

There are several possible factors that contribute to sex differences in neurodegenerative diseases. Estrogen is the most frequently discussed factor due to its neuroprotective effects. Loss of estrogen after menopause increases the risk of neurodegenerative diseases, which indicates that estrogen plays an important role in disease progression and onset. During menopause transition, the follicle-stimulating hormone (FSH) to estrogen ratio increases due to increased FSH levels and decreased estrogen levels. The FSH/estrogen ratio can be applied as a screening method for MCI in postmenopausal women [51]. In AD patients under sodium benzoate treatment, reversal of the FSH/estrogen ratio is associated with better cognition improvement [11]. FSH and the FSH receptor are found in hippocampal neurons [52], and FSH-mediated neuroestrogen generation is associated with the regulation of neuroplasticity [53].

In addition to estrogen, other neurosteroids—i.e., the steroids produced within the nervous system—are reported to have neuroprotective effects [54,55]. Circulating progesterone is decreased during menopausal transition and drops dramatically following menopause [56,57]. The neuroprotective effects of progesterone are well documented, and progesterone and its metabolites, 5α-dihydroprogesterone (5α-DHPROG) and 3α,5α-tetrahydroprogesterone (3α,5α-THPROG), decrease with aging [58]. Levels of insulin-like growth factor 1 (IGF-1), which can be regulated by estrogen, are also reported to decline in serum with aging, especially after the age of 40 years [59,60]. Interestingly, production and secretion of estrogen, progesterone, and IGF-1 by the Central Nervous System (CNS) have been also detected [61,62,63]. In this section, we will discuss the effects of estrogen and other neurosteroids on neuroprotection and the impacts of menopause on neurodegeneration.

### 3.1. Effects of Estrogen on Neuroplasticity

The association of estrogen with AD progression has been implicated in morphological changes of neuronal cells, the cholinergic system, and amyloid processing. Through cell culture systems and ovariectomies in rodents, the effects of estrogen or menopause on nerve degeneration have been well characterized. 17β-estradiol, an estrogen steroid hormone, can rapidly induce long-term potentiation (LTP) and increase the spine density of the neurons of hippocampal slices. These synaptic plasticity effects are mediated through mitogen-activated protein kinase (ERK/MAPK) pathways [64]. Through binding to estrogen receptor (ER), 17β-estradiol increases the phosphorylation of AKT and Tropomyosin receptor kinase B (TrkB), the receptor of brain-derived neurotrophic factor (BDNF), in the hippocampus [65]. As AKT and BDNF are molecules which play well-known roles in synaptic plasticity, this result indicates that 17β-estradiol and ER have the ability to regulate hippocampal synaptic plasticity and improve memory. There are several studies showing that activation ERs can increase dendritic spine density and/or cognitive performance [66]. Using selective ERβ agonists, it has been shown that estrogen increased key synaptic proteins in the hippocampus, which might affect synaptic plasticity and memory through Erβ [67]. Moreover, infusion of ERα or ERβ agonists to the hippocampus enhanced memory in ovariectomized female mice in an ERK-dependent manner [68]. The expression levels of membrane ERα in synapse are higher in female mice, and ERα is essential in 17β-estradiol-induced LTP in females, as well as ERβ in males [69]. In addition, 17β-estradiol and the G-protein-coupled estrogen receptor 1 (GPER1)-specific agonist G1 can induce BDNF release and trigger LTP [70].

The impacts of menopause on the central nervous system are mostly due to the dramatic loss of estrogen. The hypothalamic–pituitary–gonadal axis, in which endocrine and neural systems interact with each other, is disturbed during pre- and postmenopause, leading to neurodegeneration [71]. Lower 17β-estradiol concentration is related to hippocampal dysfunction and poorer performance on initial learning and memory retrieval [72,73]. Moreover, shorter 17β-estradiol exposure due to delayed age at menarche, younger age at menopause, shorter reproductive span, and hysterectomies are correlated to higher dementia risk [74]. The average density of N-methyl-d-aspartic acid receptors (NMDARs) current in dorsal root ganglia is much higher in female rats than male rats [75]. Interestingly, 17β-estradiol-increased LTP depends on NMDA receptors in females and AMPA receptors in males [76,77]. The positive effects of 17β-estradiol on the hippocampus and memory are enacted through different signal pathways in males and females. In females, 17β-estradiol enhances memory consolidation through ERK, but not in males [78], and metabotropic (mGluR1a) and ionotropic (NMDA) glutamate receptors are essential for 17β-estradiol-mediated ERK activation and memory consolidation [68,79]. However, the effects of hormone replacement therapy on preventing AD or dementia in postmenopausal women are inconsistent [80,81]. In addition, although epidemiology studies indicate that estrogen loss after menopause might trigger severe symptoms in AD, the results from hormone replacement therapy in postmenopausal AD patients are varied [82,83]. The latest meta-analysis study showed that hormone replacement therapy is correlated with reduced risk of AD. Moreover, longer duration and natural steroid formulations are linked to better protection [84]. A recent review suggested that during the time frame of MCI, typically preceding fully developed AD, hormone replacement treatment could postpone and possibly even prevent the onset of diagnosable AD [85].

### 3.2. Immunomodulatory Effects of Estrogen

In addition to reproductive and sexual functions, estrogen has been shown to be able to modulate the immune system. Most immune cells and epithelial cells express ERs and progesterone receptors, and steroid hormones regulate innate immunity against bacteria in epithelial tissues [86]. 17β-estradiol protects the epithelium through modulating the expression of inflammatory cytokines and antimicrobial proteins [87]. Treatment with 17β-estradiol in epithelial cells can reduce inflammation but increase antimicrobial molecules, which maintain the balance between inflammation-induced tissue damage and antimicrobial ability [88]. Moreover, loss of ERα on epithelial cells also impairs the vaginal structural integrity and increases leukocyte infiltration [89]. These effects on the epithelium protect the vagina, urinary tract, and respiratory tract from infectious agents [90,91,92]. In addition to antimicrobial ability, estrogen can modulate gastrointestinal epithelial secretions [93]. For instance, 17β-estradiol inhibits Cl- secretion in rat distal colonic epithelium caused by endotoxins, which may prevent diarrhea [94,95]. Thus, the gastrointestinal tract is also a sex-steroid-targeted organ.

Since estrogen can affect the GI tract and the gut–brain axis participates in the pathogenesis of neurodegenerative diseases, the impact of menopause on the gut microbiota needs to be discussed. The gut microbiota is essential in maintaining homeostasis, and changes in the gut microbiota can also lead to alteration of behavior and cognition [96]. During menopause transition and the early menopausal period, abdominal irritation was found and negatively related to estrone glucuronide levels [97]. In postmenopausal women, the urinary estrogen level is associated with fecal microbiota [98]. Soy isoflavones, which exhibit estrogen-like properties, can significantly change the fecal bacterial community in postmenopausal women [99]. In ovariectomized mice, ovariectomy was associated with a shift in the beta diversity of the gut microbiota analyzed by 16S rRNA gene sequence analysis [100]. A recent study showed that *Verrucomicrobia* and *Actinobacteria* were significantly increased in ovariectomized mice compared with a control group [101]. In addition, the reduction in microbiota diversity in ovariectomy was changed by a probiotic and soy isoflavone diet treatment [102]. Another study on ovariectomized mice demonstrated that long-term conjugated estrogens plus bazedoxifene did not change the overall cecal or fecal microbiome community but modulated gut microbial β-glucuronidase activity [103]. These results show that changes in sex steroid hormones did affect the intestinal microecological environment, which might contribute to neurodegenerative diseases.

### 3.3. The Role of Estrogen in AD

**Figure 1:**
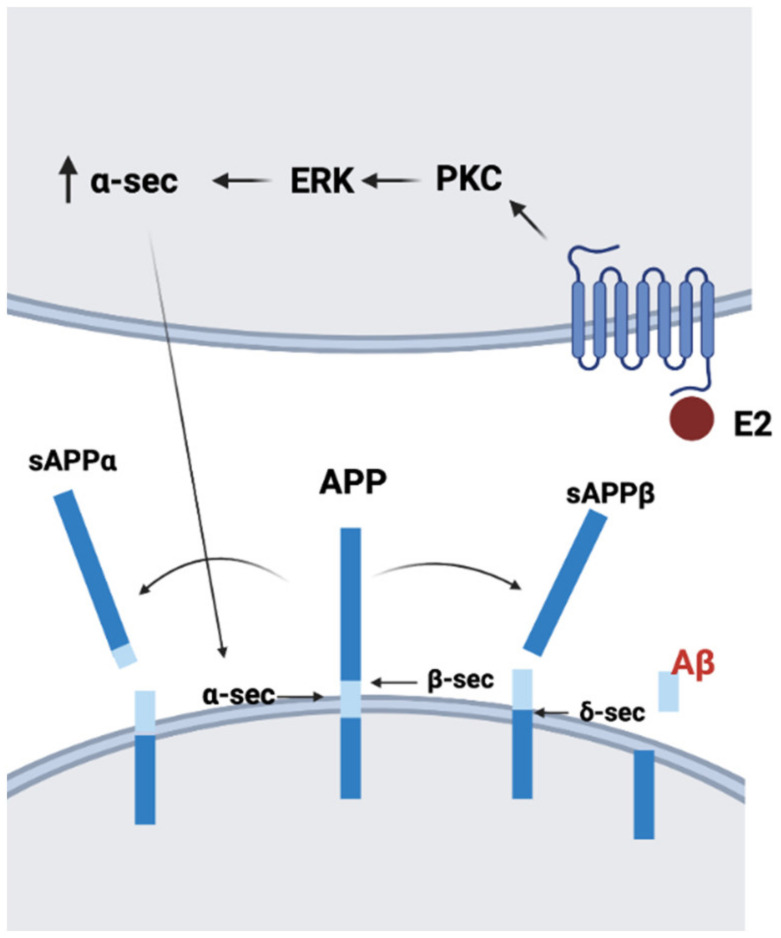
Proposed model of the mechanism of 17β-estradiol (E2) regulating amyloid precursor protein (APP) processing. E2 increases α-secretase (α-sec) via the PKC-ERK pathway and enhances sAPPα secretion (created with BioRender.com).

Neurotoxic Aβ is one of the major pathogenic agents involved in the onset and progression of AD. The amyloid precursor protein (APP), which is highly expressed in nerve cells, plays a crucial role in AD pathogenesis. Aβ is generated by APP processing. At first, APP is cleaved by β-secretase BACE-1, leading to soluble APPβ (sAPPβ) and the membrane-bound fragment β-CTF. Further processing of β-CTF by γ-secretase creates Aβ, and the depositions of Aβ induce neurotoxicity [104]. Another processing pathway of APP is mediated by α-secretase, which can generate sAPPα [105]. Interestingly, sAPPα can protect neurons against Aβ-dependent neuron toxicity [106]. Incubation with 17β-estradiol induces the release of sAPPα by the ERK pathway in mouse hippocampal HT22 cells and human neuroblastoma SK-N-MC cells [107]. A study on primary rat cortical neurons also showed that 17β-estradiol increased the secretion of sAPPα through protein kinase C [108]. These data imply that estrogen might decrease Aβ neurotoxicity by increasing sAPPα secretion (Figure 1). It is becoming more and more appreciated that viral, bacterial, and fungal brain infections can be causative for AD [109]. Similarly, it is becoming understood that generation of Aβ, including Aβ plaques, is part of the response process against such brain infections [85,110]. Interestingly, hormone replacement appears to be effective against at least several very serious infections, i.e., against the infectious agents [92,111]. Thus, the immunoenhancing effects of estrogen might also be involved in reducing abnormal Aβ accumulation.

Estrogen can regulate Aβ expression, accumulation, and degradation. A brain-selective 17β-estradiol estrogen prodrug could decrease amyloid precursor and Aβ protein levels in an APPswe/PS1dE9 double-transgenic mouse model [112]. In the same transgenic model, 17β-estradiol and its prodrug could decrease Aβ (1–40) and Aβ (1–42) levels in the brain in both intact and ovariectomized animals [113]. 17β-estradiol regulates βAPP trafficking within the late secretory pathway in neuroblastoma cells and primary neurons [114]. 17β-estradiol and progesterone increased the expression level of insulin-degrading enzyme (IDE), which is related to Aβ clearance, and this overexpression of IDE is inversely associated with soluble Aβ levels [115]. Administration of 17β-estradiol reduced Aβ accumulation and plaque formation and increased hippocampal IDE expression in 3xTg-AD mice with ovariectomy [116]. In addition to IDE, 17β-estradiol-induced matrix metalloproteinases and neprilysin are also associated to Aβ degradation [117,118]. These Aβ clearance pathways are one of the treatment strategies which might be used to remove amyloids and improve cell survival in AD [119].

Another benefit of estrogen for AD is its neuroprotective effects, and this cellular effect might delay the progression of AD. In Aβ-indued neuronal death, estrogen can protect cells against death by enhancing BcL-2, BCLxL, and heat shock protein 70 and decreasing Bax proteins [120,121,122]. In the mouse cholinergic cell line SN56, 17β-estradiol could prevent Aβ-induced cell death through estrogen-receptor-mediated pathways [123]. Treatment with estren, 4-estren-3α, and 17β-diol could activate non-genomic estradiol pathways, restored loss of cholinergic neurons, and attenuated learning deficits which were induced by Aβ (1–42) injection to mouse basal forebrain [124]. In addition, estrogen promoted the degradation of Cav1.2 through ERα in neuronal cells and in an AD mouse model, and Cav1.2 is the subunit of the L-type calcium channel, which is important in calcium overload and cell death in AD [125]. 17β-estradiol protects against oxidative stress-induced cell death in hippocampal neurons through TRPV1 [126]. In a combined bioinformatics analysis of female AD patients and AD mouse models, thioredoxin-interacting protein (TXNIP) was found specifically expressed in the hippocampus in AD. Pan et al. found that estrogen treatment protects SH-SY5Y neuroblastoma from Aβ42-induced apoptosis by increasing thioredoxin and reducing TXNIP, which is mediated by the AMPK signal pathway [127]. Since TXNIP reduces thioredoxin and increases oxidative stress, the protective effects of estrogen in AD can be linked to oxidative stress and inflammation [128].

The antioxidative properties of estrogen in neuroprotection against AD have been well discussed. Aging or ovariectomy in rodents increases mitochondrial dysfunction, which can lead to reactive oxygen species (ROS) production. This phenomenon is observed in AD animal models and patients with AD [129,130]. In aged or AD brains, not only excessive ROS but also decreased antioxidant ability contribute to neurodegeneration [131,132]. Aβ induces calcium influx, leading to mitochondrial dysfunction and oxidative stress in astrocytes, and this overproduced oxidative stress causes antioxidant molecular depletion and cell death in neurons [133]. This evidence indicates that oxidant/antioxidant imbalance can be a therapeutic target in AD [134]. 17β-estradiol induces SH-SY5Y neuroblastoma cells to express neuronal nitric oxide synthase (NOS) and manganese superoxide dismutase (MnSOD), which are linked to antioxidative and neuroprotective ability [135]. Although estrogen shows inhibitory effects on lipid peroxidation, DNA damage, and intracellular peroxide accumulation [136,137], its protective effect on Aβ-induced oxidative stress is inconclusive. Chronic incubation with Aβ increases the lipid peroxidation level, and estrogen treatment can decrease peroxidation in human SK-N-SH neuroblastoma cells by almost half [138]. In another study of human neuroblastoma SH-SY5Y cells, 17β-estradiol had little effect on Aβ-induced ROS generation [139]. Along with Aβ-induced oxidative stress in the brain, increased biomarkers of lipid peroxidation in blood were found in mild cognitive impairment (MCI) or AD patients [140,141]. Plenty of evidence shows that estrogen displays a strong antioxidant ability, and estrogen treatment providing neuroprotection by reducing lipid peroxidation is a possible therapeutic approach to slow down or prevent progression of AD [142].

Inappropriate immune response in the CNS contributes to the severity of the pathology in AD. During AD progression, resting microglia, the immune cells in the brain, are activated by Aβ deposition and tau protein [143]. A longitudinal study on microglial activation in MCI and AD subjects showed that there are two peaks of microglial activation in the trajectory [144]. The pro-inflammatory cytokines and ROS released from activated microglia contribute to neuronal damage and neurodegeneration [145]. On microglia, the presence of estrogen receptors, namely ERα, ERβ, and GPER1, has been detected [146]. 17β-estradiol inhibits inflammatory genes’ expression by reducing the transcription factor NF-κB [147]. 17β-estradiol can downregulate lipopolysaccharide-induced inducible nitric oxide synthase (iNOS) expression, and this anti-inflammatory effect is mediated by the MPAK pathway [148]. In addition to NO, estrogen inhibits cerebral ischemia-induced inflammatory cytokines such as IL-1β and TNF-α from releasing via GPER1on forebrain microglia [149]. ERα phosphorylated at Ser216 is only observed on microglia, not astrocytes, in mixed astrocyte and microglia culture or mouse brain. Stimulation of mouse brain or microglia with lipopolysaccharide induces inflammatory cytokines, and these pro-inflammatory cytokines are upregulated in the brain and in microglia bearing a phosphorylation-blocked ERα S216A mutation. These data validate that estrogen conducts anti-inflammatory action via ERα in microglia [150]. Moreover, patients with higher ERα promoter methylation rates showed decreased ERα mRNA expression and impaired cognition [151], which implies the importance of ERα in AD. As mentioned, it is becoming more and more appreciated that infection may be one causative factor for AD.

Apolipoprotein E (APOE), the strongest genetic risk factor for late-onset AD, is another key factor contributing to sex-specific differences in AD. In the brain, APOE is highly expressed on astrocytes, and APOE modulates lipid homeostasis [152]. Ratnakumar et al. compared neuronal genes regulated by estrogen in ovariectomized rhesus macaques and exome sequencing data from female AD patients and found that estrogen upregulates APOE gene expression [153]. In cultured hippocampal neurons and rat hippocampus, 17β-estradiol increased APOE mRNA and protein expression via ERα but not ERβ [154]. However, unlike rodents, which only have one APOE isoform, there are three different APOE isoforms, namely APOE2, APOE3, and APOE4, in humans. APOE4 is associated with a higher risk of AD, and people with APOE2 homozygotes present an exceptionally low likelihood of AD [155]. In transgenic mice with human APOE2, APOE3, or APOE4, Nathan et al. found that 17β-estradiol-induced APOE2 and APOE3, but not APOE4, can enhance neuritic outgrowth [156]. Thus, although estrogen can induce APOE expression, the APOE genotype may decide the outcomes of estrogen exposure.

Mitochondrial dysfunction has been associated with AD progression in pre- and postmenopausal patients. Pieces of evidence indicate that Aβ-mediated toxicity is the key factor that triggers mitochondrial dysfunction [157]. Aβ overload increases membranes’ permeabilization of calcium, and excessive calcium leads to increased mitochondrial membrane permeability, which causes apoptosis and necrosis [158]. The voltage-dependent anion channel (VDAC), which is expressed on cell membranes and mitochondria, regulates calcium homeostasis and interacts with ERs in neuronal membranes [159]. Administration of estrogen modulates VDAC phosphorylation within a few minutes and leads to a reduction in VDAC opening. This phenomenon might be another mechanism of estrogen-provided neuroprotection [160]. In addition to VDAC, ERβ on neuronal mitochondria (mtERβ) can also affect mitochondrial function. In female AD patients, mtERβ expression and mitochondrial cytochrome C oxidase activity were significantly reduced, which implies that the deficiency of mtERβ might be associated with the dysfunction of mitochondria in AD [161]. A recent study showed that mitochondrial damage, including abnormal mitochondrial function and biogenesis, occurs prior to cognitive decline in the hippocampus of ovariectomized mice [162]. Compared to a premenopausal control, post- and perimenopausal females displayed reduced platelet mitochondrial activity, which was highly correlated to cerebral glucose metabolism [163]. The same group also analyzed in vivo brain mitochondrial ATP production of female participants across the pre-, peri-, and postmenopausal phases using multi-modality neuroimaging. Their results showed that postmenopausal brains have higher mitochondrial ATP production, which was correlated with preserved cognition [164], which implies that there might be a compensatory mechanism.

### 3.4. The Role of Estrogen in PD

Women have a lower risk of PD; however, PD symptoms increase in women after menopause due to the drop in endogenous estrogen [165]. Since estrogen can improve object recognition memory [166], which depends on dopamine neurons, it is not surprising that estrogen can modulate dopamine-dependent cognition. Administration of estrogen can alter dopamine transmission in the striatum, nucleus accumbens, and prefrontal cortex [167]. However, ERα- and GPER are found in cholinergic neurons and glia, not in axons and terminals of dopamine neurons [168]. Moreover, sERα/β and GPER express mainly on pyramidal cells of the hippocampus [169,170]; thus, the improvement of memory and cognition by estrogen might not be directly related to dopamine neurons. 17β-estradiol treatment stimulates the expression of glial cell-line-derived neurotrophic factor (GDNF) and protects dopaminergic neurons from 6-hydroxydopamine toxicity [171]. Moreover, estrogen can increase circulating IGF-1 and protect postmenopausal women from PD via IGF-1 [172]. Results from a meta-analysis also showed that hormone replacement therapy in menopausal women may reduce the risk of PD [42,173]. Altogether, lack of estrogen after menopause appears to play an important role in PD development.

### 3.5. The Effects of Progesterone on Neurodegenerative Diseases

Although the neuroprotective effects of progesterone are well known, the association of progesterone and neurodegenerative diseases, especially AD, is not very clear [174]. Endogenous progesterone and 3α,5α-THPROG are decreased by Aβ administration in vivo and in vitro. Exposure to progesterone of primary neuron culture and rat brain can induce Aβ-clearance-related factor expression, especially insulin-degrading enzyme [115]. In an AD mouse model, progesterone could prevent tau hyperphosphorylation but not Aβ accumulation [175]. However, some hormone replacement therapies including estrogen plus progesterone increased risk of AD [176]. A meta-analysis of hormone replacement therapy showed that synthetic progestin reduced the protective effects of estrogen on reducing risk of AD, but natural progesterone did not [84]. 3α,5α-THPROG, a metabolite of progesterone, showed superior effects on pathophysiology, cognition, and memory in a 3xTg AD mouse model [177,178], and the underlying mechanism involved 3α,5α-THPROG regulating glucose metabolism, mitochondrial bioenergetics, and cholesterol homeostasis in the brain [179]. In contrast to the inconclusive results of progesterone treatment for PD [58], 3α,5α-THPROG showed better improved cognitive and motor functions in MPTP- or 6-OHDA-induced PD mouse models [180,181].

### 3.6. The Role of IGF-1 in Neurodegenerative Diseases

Although the IGF-1 level decreases with aging, the role of IGF-1 in menopause-related neurodegenerative diseases is still unclear. Several studies have shown decreased IGF-1 levels in blood are associated with cognitive decline or risk of AD [182,183,184,185,186,187,188,189], and some studies found increasing IGF-1 in the serum of AD patients [190,191,192]. A meta-analysis did not show a clear correlation between IGF-1 level and AD in human subjects [193]. Compared to age-matched controls, a lower CSF/plasma ratio of IGF-1 was found in AD patients [192], which implies that IGF-1 transportation from blood to brain might be important in AD. Moreover, decreased mRNA expression of IGF-1, IGF-1R, and insulin was detected in late AD cases compared to controls [194]. In early PD patients, decreased serum IGF-1 levels are correlated to poor cognition, attention, and verbal memory [195]. Using the cyclic glycine-proline (cGP)/IGF-1 ratio to represent bioactive IGF-1, it has been shown that higher IGF-1 function is correlated to better cognition in normal aged people and the PD group [196]. However, this positive correlation of IGF-1 and cognitive function is sex-dependent. A cohort study showed that IGF-1 is positively related to cognitive function measured by the Mini-Mental State Examination and Verbal Fluency test in males but not females [197]. A cross-sectional study in Ashkenazi Jews with exceptional longevity (age ≥ 95 years) showed that lower serum IGF-1 is associated with better cognitive function in females but not males [198]. These results imply that the role of IGF-1 in neurodegenerative diseases is not only sex-related but also age-dependent.

## 4. Menopause and Estrogen, Progesterone, and IGF-1 Signals

Numerous studies aimed to identify the role of estrogen, progesterone, and IGF-1 in neurodegenerative diseases. The following section will summarize the signaling pathways involved in menopause-related neurodegenerative diseases.

### 4.1. Genomic Action of Estrogen

Estrogens can mediate their neuroprotective effects in the brain through a number of mechanisms. In nature, there are three main estrogens: estriol, estrone, and 17β-estradiol. Frederiksen et al. analyzed these three estrogens’ levels throughout life and found that the 17β-estradiol level is higher than that of estrone in premenopausal women but lower than that of estrone in postmenopausal women [199]. Regarding neuroprotection in AD, all these estrogens can inhibit Aβ oligomer formation, and estriol had the strongest in vitro activity [200]. 17β-estradiol is the most potent estrogen, and the decrease in 17β-estradiol in blood during menopause contributes to the increasing risk of cardiovascular disease due to loss of cholesterol and triglyceride metabolism regulation [201]. Furthermore, cardiovascular diseases also contribute to cognitive decline [202]. The typical mechanism is via the genomic pathway, for which estrogens bind to their receptors, ERα and ERβ. ERα and ERβ are steroid nuclear hormone receptors, and they also belong to ligand-activated transcription factors [203]. After estrogens bind to ERs, the conformational change of inactive ERs induces homodimerization of the estrogen receptors and removal of the regulatory receptor-associated protein Hsp90 [204,205]. The activated ERs translocate to the nucleus and bind to estrogen response elements (EREs) on specific genes [206]. This ERE binding facilitates the recruitment of other transcription factors, leading to an increase or decrease in target genes’ expression, such as brain-derived neurotrophic factor (BDNF) and GDNF [171,207], which are important in neural protection. The responses described above are genomic responses which involve ERs and downstream signal cascades activating/repressing gene transcription. Interestingly, the response of BDNF transcription to estrogen during hippocampus development is gender- and region-dependent [208], which indicates cell-type- and gender-specific effects of estrogen on neuronal cells.

### 4.2. Non-Genomic Pathway Signals of Estrogen

The non-genomic response, also called the non-classical pathway, which does not involve ERE transcription factors, acts much faster than the genomic response. It usually responds within seconds because signals are mediated by the G-protein-coupled receptor and kinases [209]. There are four estrogen-binding membrane receptors, include G-protein-coupled estrogen receptor (GPER), Gq-coupled membrane estrogen receptor (Gq-mER), membrane subpopulation of ERs (mERα/β), and ER-X [210]. Gq-mER mainly distributes in hypothalamic proopiomelanocortin (POMC) neurons, and Gq-mER activation is related to homeostasis [211]. GPER, also named GPER1 or GPR30, is a transmembrane intracellular receptor and is found highly expressed on some breast cancer tissue and cell lines [212]. GPFER is expressed not only on cancer cells but also in the hippocampus. Immunohistochemistry staining results showed that GPER is strongly expressed in CA1 and the dentate gyrus in the hippocampus [213]. In addition, infusion of GPER agonists to the hippocampus could enhance memory in ovariectomized female mice [214]. Besides GPER, mERα/β, Erα, and ERβ localize to the plasma membrane and are found in neurons and glia [79]. Activation of mERα/β rapidly regulates neuron function in a sex-specific manner. 17β-estradiol treatment increases spontaneous excitatory postsynaptic current amplitude in female hippocampus, but not in that of males [215]. After estrogen binds to membrane estrogen receptors, it modulates neuroplasticity by activating second signals [216]. The most frequently mentioned downstream signal of GPER is extracellular signal-regulated kinase (ERK1/2) [217]. The other molecular signals which are involved in GPER pathways are phosphatidylinositol 3-kinase (PI3-K)/Akt and cAMP [218,219]. However, in an embryonic mouse hippocampal cell line, 17β-estradiol dose-dependently increased forskolin-stimulated cyclic AMP levels without activating the ERK1/2 pathway [220]. In a PD mouse model, GPER was required for ERα-induced neuroprotection on dopamine neurons [221]. In a global cerebral ischemia model, estrogen, as well as the GPER agonist G1, promoted neuronal survival through GPER activation of this pathway [213]. Similar protective effects were also found in a spinal cord injury mouse model and traumatic brain injury [222,223]. In these two studies, activation of GPER by estrogen or G1 could increase the phosphorylation of ERK and AKT, as well as increasing BDNF expression and decreasing cell apoptosis. Estrogen can increase Trkb receptor, BDNF receptor, phosphorylation, and ERK signals via GPER and can also stimulate RhoA and Rac signals via mERα/β. These signals regulate protein synthesis and actin polymerization in hippocampal neurons [224]. In addition to GPER-induced signals, GPER participates in the IGF-1-mediated neuroprotective effect [225,226]. IGF-1 receptor (IGF-1R) and GPER regulate each other through the PI3-K and ERK/MAPK signaling pathways. Interestingly, insulin-induced hypoglycemia significantly decreases GPER protein expression in A2 noradrenergic nerve cells in females but not males [227], which indicates a sex difference in GPER gene expression regulation. In addition, ERα and ERβ are also expressed in mitochondria and against mitochondrial-mediated oxidative stress via a non-genomic pathway [228], and estrogen deficiency causes mitochondrial dysfunction prior to cognitive decline in ovariectomized mice [162].

### 4.3. IGF-1 and IGF-1R Signals in AD

Among the neuroprotective factors that are affected by menopause, IGF-1 cannot be excluded. IGF-1 is involved in synaptic plasticity, neural regeneration, and cognitive function [229]. In aged rats, administration of IGF-1 can increase NMDAR2A and NMDAR2B protein expression, which is decreased in the hippocampus of aged rats [230]. Restoration of IGF-1 levels in an adult rat brain significantly restores the age-dependent reduction in neurogenesis in the dentate subgranular proliferative zone [231]. In peri- and postmenopausal women, serum levels of IGF-1 were significantly reduced compared with the premenopausal group [60]. In an APPSwe/PS1ΔE9 AD mouse model, decreased serum IGF-1 levels were associated with early Aβ deposition in the brain [232]. Systemic slow-release formulation of IGF-1 significantly rescued Aβ accumulation and memory decline in AD rodent models [233,234]. On the contrary, the role of IGF-1R in AD is different to that of IGF-1. Through IGF1RKO conditional mutant mice and an APP/PS1 AD mouse model, George et al. showed that reduction in neuronal IGF-1R prevents Aβ-induced cognitive deficits and inflammation, which indicates that suppression of IGF-1R signaling can improve AD progression [235]. The same group also showed that IGF-1R knockout in adult APP/PS1 AD mice results in improved spatial memory and lower accumulation of Aβ-containing autophagic vacuoles [236]. Treatment with picropodophyllin, an IGF-1R inhibitor, attenuated insoluble Aβ and microgliosis in the hippocampi of AβPP/PS1 mice [237]. IGF-1R can form hybrid receptors with insulin receptor (IR), and insulin resistance is one of risk factors of neurodegenerative diseases. Thus, the interactions between IGF-1, IGF-1R, and IR form a more complex signaling pathway. The controversial results from therapeutic strategies targeting modulation of IGF-1 or IGF-1R alone indicate that this may not be an appropriate therapy for AD patients with diabetes or insulin resistance. On the other hand, one-month administration of IGF-1 by subcutaneous minipump had no effects on Aβ protein level, plaque pathology, or phospho-tau expression in a Tg2576 AD mouse model [238]. A recent study showed that adenovirus-mediated transduction of IGF-1 protected mice from Aβ-induced memory impairment [239]. Although some studies in AD animal models show that IGF-1 treatment reduces Aβ accumulation and improves behavioral and pathological AD, the therapeutic strategy targeting increasing circulating IGF-1 failed to delay AD progression in a clinical trial [240]. In agreement with the clinical trial, increasing IGF-1 by MK-677 in 5xFAD AD mice failed to prevent hippocampal Aβ deposition and other AD pathogenesis [241].

### 4.4. Progesterone Signals and Neuroprotection

Similar to estrogen, progesterone can act through classical and non-classical pathways. In the classical pathway, progesterone binds to nuclear progesterone receptors (PGRs) and stimulates gene expression. In the non-classical pathway, progesterone can bind progesterone membrane receptors (mPRs) or the membrane-associated protein progesterone receptor-membrane component 1 (PGRMC1), leading to faster responses. In addition, progesterone can be converted to 3α,5α-THPROG, which can modulate GABA receptors [242]. Progesterone attenuates Aβ-induced primary cortical neuron apoptosis by inhibiting the mitochondria-associated apoptotic pathway via PGRMC1-mediated JNK inactivation [243]. 3α,5α-THPROG can reduce Aβ-induced ERK phosphorylation in SH-SY5Y human female neuroblastoma cells in a GABA-receptor-independent manner [55]. The neuroprotective effects of progesterone and 3α,5α-THPROG are also related to anti-inflammation and myelin repair. 3α,5α-THPROG can change microglia morphology and inhibit oligodendrocyte phagocytosis via microglia [244]. These results explain the protective effects of progesterone on demyelination and oligodendrocyte degeneration in the cuprizone-induced demyelination mouse model [245]. Progesterone and its synthetic derivative nestorone were also shown to promote myelin regeneration in chronic demyelinating lesions [246,247]. Since alterations in myelination are correlated to AD and MCI [248], the AD preventive ability of progesterone, 3α,5α-THPROG, and nestorone might be due to their protective effects on myelination. Progesterone and 3α,5α-THPROG also exhibit an anti-inflammatory ability for neuroprotection [249]. Progesterone treatment induced microglia phenotype change from pro-inflammatory M1 to anti-inflammatory M2-type in a demyelination mouse model [250]. Progesterone can also reduce lipopolysaccharide-induced microglia activation in brain [251]. In MPTP-induced PD mouse models, five-day administration of progesterone could reverse MPTP-induced striatal glial fibrillary acidic protein (GFAP) overexpression, which is the marker of activated astrocytes [252]. Results from the same paper also show that progesterone increased the BDNF level. In human Schwann cell-like cells which are differentiated from adipose stem cells, progesterone and mPRα agonist activate mPRα and increase the BDNF level, in which the Src and PI3K-Akt signaling pathways are involved [253]. Although the neuroprotective effects of progesterone and its associate neurosteroids are clear, continued treatment with progesterone or medroxyprogesterone acetate had no benefit, or even a negative impact, on memory in surgically menopausal rats [254]. This indicates that simply activating progesterone signals is not sufficient to treat AD. In addition, the age-associated changes in sensitivity of the brain to progesterone create “therapeutic time windows” for hormone replacement therapy. This issue has been discussed thoroughly in several reviews [254,255].

### 4.5. Estrogen, IGF-1, and IGF-1R Signals

**Figure 2:**
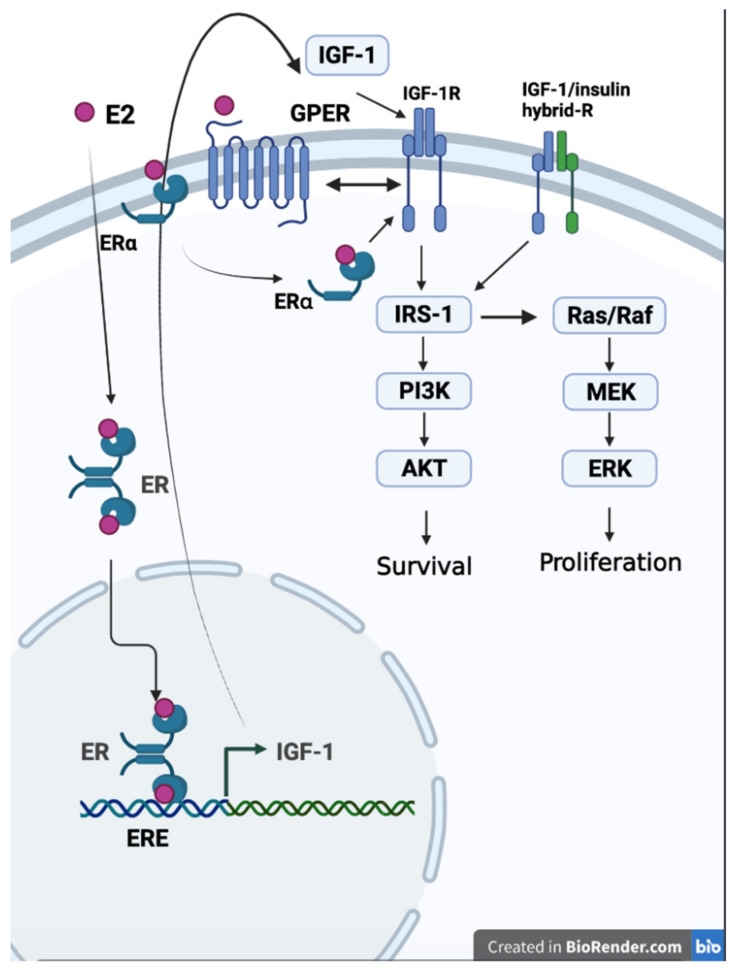
The correlation of estrogen, estrogen receptors, IGF-1, and IGF-1R. Estrogen (E2) increases IGF-1 gene expression through genomic pathways and enhances IGF-1 receptor activity through non-genomic pathways, including mER and GPER (created with BioRender.com).

Through in vitro studies, the neuroprotective effects of the estrogen/IGF-1/IGF-1R axis have been well characterized. Estrogen enhances IGF-1 transcription via the genomic pathway, which induces ER nucleus translocation and binds to ERE on the IGF-1 promoter [256]. Increased IGF-1 levels expand IGF-1R signals. Estrogen can also increase IGF-1R activity through non-genomic pathways. IGF-1-mediated cell proliferation requires GPER, and GPER transcription is also enhanced by the IGF-IR/PKCδ/ERK pathway [257]. In addition, ERα can also affect IGF-1R function [258,259]. Downstream of IGF-1R, the PI3K/Akt and Ras/MEK/ERK pathways are most frequently mentioned. These signals stimulate cell survival and proliferation. Moreover, IGF-1R activation can increase BDNF expression, which plays an important role in cognition [260]. Increased IGF-1 or IGF-1R activity can promote hippocampal progenitor cell proliferation [261], and lack of IGF-1 leads to a smaller hippocampal volume [262]. In addition, IGF-1R signals seem to interfere with Aβ clearance. Reduced IGF signaling in AD mice can protect mice from AD pathogenesis, which is associated with less hyperaggregation of Aβ in the brain [236,263]. As the disease progression is difficult to reverse, including Aβ accumulation formed in the late AD phase, the ineffectiveness of potent inducers of IGF-1 on AD patients might be due to the negative effects of IGF-1R on Aβ clearance. Considering that oxidative stress and glia-mediated inflammation also contribute to neurodegenerative diseases, the anti-inflammatory effects of IGF-1/IGF-1R still cannot be ruled out. Although IGF-1 suppresses MPTP/MPP +-induced astrocyte activation via GCER [225,264], the IGF-1R inhibitor prevented neuroinflammation in the hippocampus in an AD mouse model [237]. Since MPTP/MPP induced dopaminergic neurotoxicity in a PD model, these controversial results imply that IGF-1/IGF-1R signals might play different roles in PD compared to AD. Figure 2 illustrates the interaction of estrogen, estrogen receptors, IGF-1, and IGF-1R signals.

## 5. Estrogen and NMDA Receptor Signals in Neurodegenerative Diseases

Estrogen is well known to influence cognition by regulating synapse structure and function. On CA1 pyramidal cells in the hippocampus of adult female rats, 17β-estradiol can increase dendritic spine density and excitatory synapses [14]. In addition, 17β-estradiol treatment increased the LTP magnitude in CA1, which involved NMDA receptors [265]. In CA1 pyramidal neurons, which are innervated by the temporoammonic pathway, 17β-estradiol induces LTP via ERα and PI 3-kinase signaling, and this ERα-induced LTP is NMDAR-dependent [266]. By using NMDA receptor antagonists and cAMP inhibitors, a study in mice showed that estrogen improves object memory through NMDA receptors and PKA activation within the dorsal hippocampus [267]. In ovariectomized rats, 17β-estradiol enhanced hippocampal LTP and novel object recognition via NMDA receptors that contained NR2B, and these enhancements could be reduced by the NR2B subunit antagonist Ro25-6981 [268]. However, 17β-estradiol treatment did not alter the protein expression level of NMDA receptor subunits, including NR1, NR2A, and NR2B [269]. The possible mechanism by which 17β-estradiol improves memory and cognition might include phosphorylation and recruitment of NR2B-containing NMDARs to synapses. In addition, a study in the dentate gyrus of juvenile male rats showed that low and high doses of 17β-estradiol had opposite effects on NMDA receptors. The bidirectional effect on NMDA receptors due to the different doses of 17β-estradiol implies activating ERα or ERβ as their downstream signaling [270]. Thus, dosage of 17β-estradiol should be considered, as high and low dosages result in vastly different effects.

**Figure 3:**
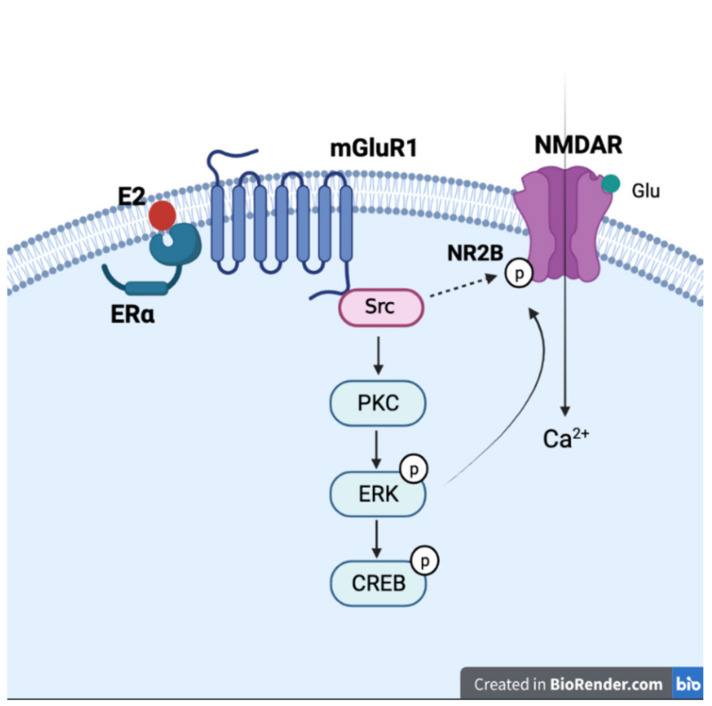
Interaction of estrogen and NMDA receptor with mGluR1 and signaling molecules. General description of the proposed mechanism of how estrogen activates mGluR1 through mERα and leads to activation of NMDA receptor by Src and other second messenger signaling cascades (created with BioRender.com).

Estrogen may also influence NMDA receptor signals through interaction with metabotropic glutamate receptors (mGluRs). In CA3-CA1 hippocampal pyramidal neurons, 17β-estradiol activates ERα and regulates CREB phosphorylation via activation of mGluRs [271]. Treating the dorsal hippocampus with ERα and ERβ agonists also enhanced novel object recognition and object placement memory via ERK signals in ovariectomized mice, and the 17β-estradiol-improved novel object recognition could be reduced by the mGluR1 antagonist [68]. Since ERα, ERβ, mGluR1, and ERK gather at specialized membrane microdomains, the 17β-estradiol-induced enhancement might be the consequence of ER/mGluR complex formation and downstream signaling. Moreover, activation of mGluR1 reinforces NMDA current through Src kinases [69,272], and 17β-estradiol is reported to be able to increase NR2B phosphorylation through Src signaling [273]. The depressive-like phenotype and reduced neurogenesis of male mice lacking sigma-1 receptor can be rescued by 17β-estradiol treatment. These neuroprotective and antidepressant effects of 17β-estradiol are mediated by Src and NR2B [274]. These results indicate that estrogen might enhance NMDA signals via the mGluR1 and Src pathways, and these signals participate in estrogen-induced neurogenesis [273] (Figure 3).

**Figure 4:**
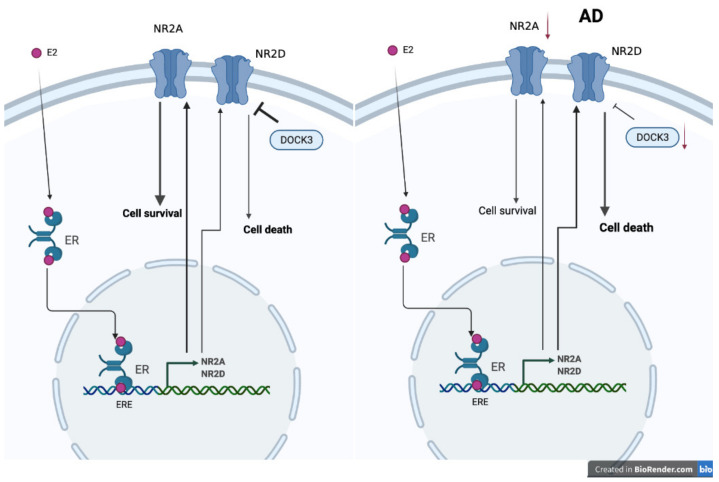
Proposed mechanism of why estrogen treatments fail in promoting neuron survival via NMDA receptors in AD patients (created with BioRender.com).

Although cell and animal studies all show estrogen can provide neuroprotective effects via NMDARs [275], randomized clinical trials of hormone replace therapy on female AD patients show no improvements in cognitive symptoms [276]. One possibility is the imbalance of NMDARs and signal molecular expression at the postmenopause stage and under estrogen stimulation. The NMDA receptor type 2D (NR2D) gene, which contains estrogen response elements (EREs), is a target for estrogen signals in the brain [277]. Indeed, NR2D mRNA expression is decreased in ovariectomized mice and can be restored by estrogen [278]. In AD patients, as well as in aging mice, a decreased expression level of NR2A and NR2B, but not NR2D, has been noted [279,280,281]. Unlike NR2A, more related to neuroprotection, NR2D overexpression induces neuronal death [282]. Dedicator of Cytokinesis 3 (DOCK3), which is decreased in the brains of AD patients, can protect neurons from NR2D- and NR2B-induced excitotoxicity [283,284,285]. Although estrogen can prevent NR2B-induced neural cytotoxicity via the GPR30/ERK signal pathway [286], it might also increase neuronal death by increasing NR2D expression. Moreover, there is no evidence showing that estrogen can modulate DOCK3 expression. Thus, the failure of hormone replacement therapy in AD patients might be due to the unwanted excessive NR2D expression but no equally inhibitory signal from DOCK3 (Figure 4).

## 6. Other Potential Therapeutic Strategies

### 6.1. Effects of Phytoestrogens on AD

In addition to classic hormone replacement therapy, the use of phytoestrogens in women with menopause can help reduce symptoms. Phytoestrogens, having a similar structure to 17β-estradiol, are plant-based compounds, including isoflavones, lignans, cumestan, and lactones [287]. Among these phytoestrogens, isoflavones have the strongest estrogenicity [288] and have been proposed as synthetic selective estrogen modulators [289]. A meta-analysis study showed that isoflavone supplementation may improve cognition in postmenopausal women regardless of methodological flaws [290]. Isoflavones have shown their neuroprotective, antioxidant, and anti-inflammatory effects in several animal models [291,292,293,294]. Moreover, isoflavones can improve cognition and reduce Aβ and tau levels in cell and rodent AD models [295,296,297,298]. Genistein, an isoflavone, is able to reduce Aβ-induced neurotoxicity and oxidative stress through various intracellular signals, including the ERK/MAPK pathway [299]. A recent study showed that polyhydroxyisoflavones can serve as a scaffold that prevents Aβ and tau aggregation [300]. Compared to estrogen, the binding ability of phytoestrogens to ERs is weak, and some phytoestrogens have a higher binding affinity for Erβ, which can inhibit proliferation in breast cancer [301]. Thus, phytoestrogens seem to have similar neuroprotective effects as estrogen but less carcinogenic potential. However, more studies are needed for evaluating the risks and therapeutic effects of phytoestrogens on preventing or treating AD.

### 6.2. Genomic and Non-Genomic Aspects of AD

The exact cause of Alzheimer’s disease is not clear, but both genetic and non-genetic factors contribute to the etiopathogenesis of AD. The genetic factors of early-onset AD are well studied, and mutations of the APP, PSEN1, and PSEN2 genes are the main cause of early-onset AD [302]. These genes mutation result in the Aβ1-42 peptide overproduction, which is connected to γ-secretase activity [303].

Genetic factors play important roles not only in early-onset AD but also in late-onset AD. One or two APOE ε4 alleles increase the risk of late-onset AD [304,305]. Carrying the APOE ε4 allele is associated with increasing Aβ accumulation and enhanced neuroinflammation [306]. In addition to APOE ε4, several gene variants, including BIN1, ABC7, PICALM, MS4A4E/MS4A6A, CD2Ap, CD33, EPHA1, CLU, CR1, and SORL1, were also found to be associated with Aβ accumulation and linked to late-onset AD [307]. The variants of estrogen metabolic-related genes, such as estrogen receptor β gene (ESR2), cytochrome P450 19A1 gene (CYP19A1), and cytochrome P450 11A1 gene (CYP11A1), are also associated with AD risk [308]. Through meta-analysis, polymorphisms of the CYP19A1 gene were found to be significantly correlated with increasing AD susceptibility [309]. To identify more genomic variants contributing to AD, several genome-wide association studies (GWASs) have been conducted to identify multiple susceptibility loci [310], and deep learning models can be used for detecting AD through the findings of GWASs [311]. These results suggest that combing GWAS on AD and menopause may identify novel pathways for therapeutic targets.

Although genomic factors are inherited and fixed, non-genomic factors associated with the onset and progression of AD may offer chances for treatment. The non-genomic factors contributing to AD risk include head injury in males, age, diabetes mellitus, conjugated equine estrogen use with medroxyprogesterone acetate, current smoking, and lower social engagement [312]. Estrogen and progesterone have neuroprotective effects on head injury [223], and the changes in sex hormones at menopause may increase diabetes risk as well [313]. Moreover, menopause and diabetes mellitus are well-known risk factors of cardiovascular diseases, and several cardiovascular diseases are related to dementia [314]. Thus, interventions targeted to menopause may also reduce non-genomic factors and enable prevention and treatment of AD.

## 7. Conclusions

There are growing lines of evidence correlating menopause with risk and progression of neurodegenerative diseases. In this review, we summarized the association of estrogen, progesterone, IGF-1, and their signal pathways with AD and PD. Based on the current literature, decreases in estrogen, progesterone, and IGF-1 increase inflammation, impair Aβ clearance, and hinder their neuroprotective effects. Not all clinical trials of hormone replacement or IGF-1R potentiator showed convincing benefits in AD patients. The modulation of estrogen, progesterone, or IGF-1 levels alone in aging populations may not be sufficient to reverse disease progression due to the complex signal pathways in neurodegenerative diseases. While diagnosed AD may not be consistently treatable by hormone replacement, its preceding stage entailing MCI may be. The combination of estrogens, IGF-1, or other neurotrophic factors in specific time windows, such as in the phase of MCI preceding fully developed AD, may provide a therapeutic strategy in postmenopausal AD patients.
